# Listening to Those at the Frontline: Patient and Healthcare Personnel Perspectives on Tuberculosis Treatment Barriers and Facilitators in High TB Burden Regions of Argentina

**DOI:** 10.1155/2014/135823

**Published:** 2014-09-28

**Authors:** Sarah J. Iribarren, Fernando Rubinstein, Vilda Discacciati, Patricia F. Pearce

**Affiliations:** ^1^School of Nursing, Columbia University, 630 West 168 Street, New York, NY 10032, USA; ^2^Institute for Clinical Effectiveness and Healthcare Policy, Dr. Emilio Ravignani 2024, C1414CPT Buenos Aires, Argentina; ^3^Division of Family and Community Medicine, Hospital Italiano de Buenos Aires, Juan D. Perón 4190, C1181ACH Buenos Aires, Argentina; ^4^School of Nursing, Loyola University, 6363 Saint Charles Avenue, Stallings Hall, New Orleans, LA 70118, USA

## Abstract

*Purpose*. In Argentina, tuberculosis (TB) control measures have not achieved key treatment targets. The purpose of this study was to identify modes of treatment delivery and explore patient and healthcare personnel perceptions of barriers and facilitators to treatment success. *Methods*. We used semistructured group and individual interviews for this descriptive qualitative study. Eight high burden municipalities were purposively selected. Patients in treatment for active TB (*n* = 16), multidisciplinary TB team members (*n* = 26), and TB program directors (*n* = 12) at local, municipal, regional, and national levels were interviewed. Interviews were recorded, transcribed verbatim, and analyzed using thematic analysis. *Results*. Modes of treatment delivery varied across municipalities and types of healthcare facility and were highly negotiated with patients. Self-administration of treatment was common in hospital-based and some community clinics. Barriers to TB treatment success were concentrated at the system level. This level relied heavily on individual personal commitment, and many system facilitators were operating in isolation or in limited settings. *Conclusions*. We outline experiences and perspectives of the facilitating and challenging factors at the individual, structural, social, and organizational levels. Establishing strong patient-healthcare personnel relationships, responding to patient needs, capitalizing on community resources, and maximizing established decentralized system could mitigate some of the barriers.

## 1. Introduction

Despite progress in treatment and prevention, tuberculosis (TB) remains a major global public health problem, particularly in low- and middle-income countries [1, 2]. The World Health Organization recommended Directly Observed Treatment, Short-Course (DOTS) strategy includes five key components: securing political commitment, strengthening detection and diagnosis, ensuring drug availability, monitoring outcomes, and providing directly observed therapy (DOT) [3]. Although DOTS has been widely adopted and has contributed to TB control progress, TB case rates in many countries are either stagnant or decreasing more slowly than expected, possibly due to incomplete application of effective control measures and care [4]. DOTS was implemented in Argentina in 1996, but overall TB treatment success rates have varied little, and there has been no significant improvement over the past 10 years [5].

Treatment success is defined as either the completion of treatment (without bacteriological confirmation) or cure (negative sputum smear at 6 months and at least once prior to 6 months) [6]. In Argentina, success rates have ranged from 48 to 66% for new sputum smear positive over the last 12 years [7], and the last country report (2011/2012) indicates that nearly 45% of new pulmonary TB cases had no final treatment outcome documented [8]. The cause of high rates of loss to follow-up is unclear, other than likely inefficiency in collection of data. Of more than 11,000 new cases identified each year, less than 50% receive treatment by DOT [5]. Specifically in the province of Buenos Aires, with the highest concentration of TB cases, DOT was the reported mode of treatment delivery for only 20% of the documented cases [9]. It is not well understood why DOT is being applied at such low rates or what other modes of treatment delivery are commonly being applied.

Nonadherence to TB therapy can lead to poor health outcomes, such as prolonged infectivity, increase in risk of relapse after treatment, generation and propagation of drug resistance, treatment failure, and increased mortality, all of which pose a serious health risk for individuals and communities [10, 11]. Treatment adherence is considered a primary determinant and a proxy for treatment success, yet not the only requirement for an efficient program [12, 13]. Barriers to treatment completion have been described as an interaction among structural, personal, and organizational factors within a social context [14]. Factors identified as barriers include limited access to healthcare, stigma attached to the disease, quality of medications, drug resistance, and patients' immunity and metabolic capabilities [2, 15].

In Argentina, all TB treatment care, including medication, is provided free of charge in the public sector. The National TB Program (NTP) is responsible for all drug provision and patient monitoring. The National Institute of Respiratory Diseases (ANLIS-Coni) in Argentina recommends an exchange of experiences and methods of success among regions in order to spread successful strategies to other parts of the country [5]. Although investigators in Argentina have reported on TB trends, patterns of resistance, delays in diagnosis and treatment, and outcomes applying DOT [16–21], the experiences and perspectives of patients and healthcare personnel dedicated to TB control have not been previously explored. Qualitative, in-depth inquiry can help to understand a phenomenon to gain insight into the problem and to provide a foundation upon which to identify appropriate solutions [22].

This study served as a foundational study to a prospective cohort trial currently in progress (patient and system factors associated with successful treatment of tuberculosis (1R01AI083229-01)). Specific study aims were to identify modes of treatment delivery and explore perceived barriers to and facilitators of treatment success from the perspective of patients being treated for active TB, multidisciplinary TB team members, and TB program directors at local, municipal, regional, and national levels.

## 2. Methods

### 2.1. Study Design

The study design was descriptive qualitative, using semistructured, face-to-face group and in-depth individual interviews [23, 24]. The theoretical framework to guide research and data analysis was adapted from the treatment adherence model developed by Munro et al. [14]. Factors were organized into the following categories:* individual* (e.g., patient and healthcare worker's individual characteristics, responses, beliefs, or actions);* structural/social* (e.g., factors over which the patient has little control, such as economic, political, cultural, or environmental factors, discrimination and inequality, gender norms, stigma, or family/community support); and* healthcare services or organizational* (e.g., healthcare center characteristics, system coordination, programs, and data management). The model was selected as an initial organizational and theoretical tool for two reasons: first because treatment adherence is recognized as a major factor in treatment success and second because we believe the model incorporates the complex nature of interactions and levels of factors that influence treatment success. The five key components of DOTS were also considered during analysis [3].

Ethical approval was granted by the Comité de Ética de Protocolos de Investigación (Research Protocol Ethics Committee) of Hospital Italiano, Buenos Aires, Argentina. All participants provided written informed consent.

### 2.2. Setting and Participants

The study setting was focused in Health Regions V and VI, located in the province of Buenos Aires. Region V is a large geographic region serving a population of 3.5 million and is comprised of 13 municipalities, each responsible for between 15 and 25 local primary healthcare centers. The region accounted for one-third of the TB cases (incidence 49.2/100,000) within the province of Buenos Aires [25]. Treatment success rates within municipalities in Health Region V range from 33% to 90% (Region V annual report). In Region VI, success rates ranged from 40 to 66%, with an average default (treatment abandonment) rate of 30% over the last 3 years [8].

A sequenced, purposive sampling design was used. Based on historic treatment outcomes and the regional director's recommendation, nine municipalities were selected from Health Regions V and VI. We included municipalities with historically high and low success rates in order to capture perspectives of what factors improved treatment success and what factors appeared to impede it. From within the nine municipalities, patients undergoing TB treatment and their family members (*N* = 16, comprised of 10 patients and six family members) and multidisciplinary healthcare team members (*N* = 38, comprised of TB program directors (national (*n* = 1), regional (*n* = 2), and municipal (*n* = 8) levels), assistant directors (*n* = 3), physicians (*n* = 6), nurses (*n* = 5), social workers (*n* = 6), and community health promoters (*n* = 7)) were interviewed. The district directors reported on average more than 10 years of work experience at the district level, and the other healthcare team members reported from 1 to 17 years. Recruitment of patients and any family members was done by healthcare personnel in the clinics. The healthcare personnel were asked to identify patients undergoing TB treatment or who had abandoned treatment. Patients and family members ranged from 7 to 48 years of age; nine were females. Patients were reimbursed for their time with a $25 USD equivalent voucher for groceries at a local store or provided with basic staple groceries where vouchers were unavailable at the local stores.

### 2.3. Data Collection and Analysis

We held nine group interviews. Interviews were conducted separately for provider (*n* = 6) and patient/family groups (*n* = 3). Individual interviews (*n* = 7) were conducted with providers when they were unable to participate in group interviews or worked independently (e.g., regional and national directors). Interviews were conducted in Spanish, assisted by a semistructured interview guide specifically designed for the study, and moderated by an experienced qualitative researcher (VD). The setting for all interviews was in healthcare centers within the selected municipality or in directors' offices. The semistructured interview guides were developed based on the previously described treatment adherence model [14] and were adapted for patients/families, healthcare teams, and directors. The interview guides focused on program characteristics, general experiences of services, characteristics that favor treatment completion, and challenges and solutions for each group. Field notes were also taken to concurrently highlight themes and to help modify guides to fill gaps where further exploration was needed. The researchers also participated in a day-long regional TB workshop to understand the overall system function.

All interviews were audio-recorded (average = 1 hour, range = 18–127 minutes) and transcribed verbatim in Spanish [26]. Transcripts were uploaded into ATLAS.ti version 6 (GmbH, Berlin, 2009) to assist with data management. Using principles of thematic analysis [23, 24, 27], the transcripts and notes were coded independently by authors SI and VD with an in vivo, open, and selective coding base on the treatment adherence model [14]. Overall coding and analysis were conducted in Spanish and later translated into English (three of the four authors—SI, FR, and VD—are fluent in both languages). Preliminary coding of barriers and facilitators was organized in a matrix from each perspective (patient, healthcare personnel, and directors) at each of the levels (individual, structural/social, and organizational). Then overarching themes from all perspectives were generated for each level. Final analysis with all authors, the report, and thematic generation were conducted in English.

## 3. Results

We identified varying modes of TB treatment delivery and several perceived barriers and facilitators to TB treatment success. We classified our findings from all participants into individual, structural/social, and organizational factors (Table 1). The modes of treatment delivery varied within healthcare centers and across healthcare facilities and were reported as frequently negotiated. The majority of barriers to treatment success were classified at the organizational/healthcare delivery system level. Interventions considered potential facilitators were not implemented system wide. That is, the degree to which these interventions were implemented was limited to one site or to a few within a municipality. In general, patients and healthcare personnel initially indicated that the system in place* functioned well* but immediately qualified “functioned well” with descriptions of the multiple challenges to TB management and achieving treatment success. We identified that, depending on context, some of the barriers and facilitators were not categorically fixed (a particular issue can have facilitative characteristics, while simultaneously having barrier characteristics) and thus are fluid in categorical terms. For example, described further below,* DOT* and* feeling better*  were both a facilitator and a barrier from the perspectives of both personnel and patients/family members. Direct quotes from patients and healthcare personnel (translated to English from Spanish) are provided as examples to illustrate some of the findings.

### 3.1. Modes of Treatment Delivery

Most healthcare centers reported providing daily DOT. However, DOT was also reported as being* negotiated* or* tailored* to meet patients' needs. In order to maintain patients who were threatening to default or who were faced with challenges to attend clinic daily and as an incentive for compliance, the number of directly observed days was negotiated, for example, 2-3 days per week, once per week, or twice monthly. One municipality offered only self-administration centralized at one healthcare center. Patients diagnosed and treated at hospitals received treatment by self-administration and were requested to return to the hospital monthly for follow-up visits and for their 1-month supply of medication. Directors and healthcare team members indicated that patients initiating treatment at a hospital would rarely be referred to a healthcare center closer to where they live or patients may be asked and decline this option.

*“There are patients who come to take their medication and there are patients who are given medication weekly and come every week to collect it.” [Healthcare personnel, FG]*

*“Well, here in Region [number removed] we have supervised treatment starting point…closer to home. The treatment is daily. Sometimes concessions can be made for the issue of distance or because there are healthcare centers that have reduced hours of operations, not like this, which is open 24 hours…” [Municipal director, FG]*

### 3.2. Individual Factors

Individual barriers and facilitators included factors related to individual and healthcare professional TB knowledge, personal experiences and relationships, the treatment regimen and its effects, and individual characteristics of patients or healthcare providers.

#### 3.2.1. Barriers

*A Lack of TB Knowledge.* Treatment seeking and continuation was described as being impacted by an initial reaction of* fear*, “going to die,” fear for family due to loss of work, and stigma related to diagnosis. Fear of TB diagnosis and potential consequences such as social stigma were both augmented and mitigated by knowing someone who had been diagnosed with TB and who had either successfully completed treatment or died from TB. Patients expressed their initial concern about what others in their community would think about them and their families.

*“That is to say, people think that it [TB] does not have a cure.” [Patient, FG]*

*“…that there was not a cure, that you would die. This is what one thinks….I realized and at the same time felt bad because he [son] was admitted [to hospital]. I cried all day. What will they say about my son? What am I going to do? On one hand we discriminate ourselves…but later I realized that it wasn't like that.” [Family member of patient, FG]*

*Drug Side Effects*. There are many pills to take and there can be side effects. Patients described treatment challenges to continuing treatment due to medication side effects and personal situations.

*“Before they [medication] caused nausea. I couldn't eat because all that I ate I threw up and I went to the clinician, but I he couldn't take away the medication because I had to continue. I just wanted to say enough but I knew…I tried to continue. I talked with the clinician and he told me I couldn't abandon the treatment because it [TB] would come back worse.” [Patient, FG]*

*“He didn't want to come any more because the pills made him feel bad, the injections too. He could not take more also because he said it hurt his buttock.” [Patient/family member, FG]*

*Interpretation of feeling better* (e.g., weight gain, return of strength, and increased energy) was identified as both a barrier and a facilitator. Patients/family members and healthcare team members described patients as interpreting feeling better as meaning “cured,” leading to treatment abandonment even with encouragement to continue from family and healthcare personnel; or patients recognized feeling better as treatment effectiveness and needing to continue treatment.

*“Well, he [brother] took everything [medication] and was doing well. After he felt better he stopped, abandoned everything. He thought that he was cured….He said he didn't need it [medication] that he was already better and was not going to continue [treatment].” [Patient/family member, brother died of TB]*

#### 3.2.2. Facilitators

*Establishing a Strong Patient-Healthcare Personnel Relationship.* A key factor contributing to treatment success for both patients and healthcare personnel was establishing a strong patient-healthcare personnel relationship. A strong relationship was considered to facilitate provision of education and be based on respect and the ability to humanize the disease and the situation. Healthcare personnel and patients also need to possess certain individual characteristics. For example, healthcare personnel needed to be committed, compassionate, supportive, and trustworthy and have a personal calling to serve others. Patients needed to be committed to being cured. There was a perception by some healthcare personnel that if the patient lacked commitment, the problem was with the patient and not the system.

*“The most effective way to influence the patient is by having a good doctor-nurse-patient relationship, and they [patients] understand that we are working to better their health and when we suggest something it is for the best.” [Healthcare personnel, FG]*

* “One must use a lot of kindness, in everything but especially this. With this type of disease [TB] we have to provide love, respect, and this at times people do not have. It is fundamental to our work to try to provide consciousness at all levels.” [Healthcare personnel, FG]*

*“It is the determination of the individual and this is what gets you to cure.” [Patient, FG]*

*“The patient that completes DOT also completes treatment in their house. The problem is with the patient, not the system.” [Municipal director, individual interview]*

### 3.3. Structural/Social Factors

Structural/social barriers and facilitators included factors related to access to care, TB awareness within the community at large, vulnerable populations, support, stigma, and political commitment to the TB program.

#### 3.3.1. Barriers

*Lack of Community TB Education in Communities and Schools.* Participants cited a lack of TB awareness in the community at large regarding etiology and treatment and a lack of acknowledgement of the gravity of TB within their communities and specifically within schools. Because of the lack of awareness by communities and healthcare personnel, a low index to suspect TB given certain symptoms was noted, which delayed TB diagnosis. Patients described having symptomatic respiratory complaints that resulted in multiple healthcare visits and misdiagnosis (e.g., pneumonia), and healthcare personnel acknowledged that such misdiagnosis often occurs in some settings. A district coordinator estimated that two to three clinic evaluations occurred for persistent symptoms until TB was suspected and testing provided. In addition, a municipal director reported that lab result notification of 15 days or more further compounded delays in treatment.

*“He [son] was vomiting blood. Well, they took him to the hospital the night I was working and when I arrived from work. …we were sent back. They took him to another hospital [name removed] and we were told the same. They didn't take an X-ray, they didn't do an analysis of the blood, nothing in the emergency…they said since he does drugs.” [Mother/prior patient, FG]*

*“In schools what I see is that TB is not talked about. They talk a lot about HIV, syphilis, sexually transmitted diseases, and maybe TB or pertussis because there was some last year. It [TB] is not taken seriously.” [Patient, FG]*

*“The official agencies put more importance on other things and tuberculosis is a disease which we live with and there needs to be more information.” [Municipal director, individual interview]*

*“…There are little children with facemasks and adults as the whole family is infected and they also are discriminated against…It signals that they are sick, you shouldn't get close, it's contagious. Therefore there is information, and lack of information.” [Director, FG]*

*Vulnerable Populations*. Specific populations were perceived as especially vulnerable to treatment success challenges. These populations included those with comorbidities (e.g., drug or alcohol addiction, HIV/AIDS), those for whom employment was informal (e.g., day labor), those considered to be part of a “health tour,” adolescents, and those living in poverty in general. Healthcare personnel recognized that loss of work opportunity due to attending daily clinic required for DOT equated to loss of financial ability to care for family. Those considered part of a “health tour” or considered to be outside of the established healthcare system were described as providing false contact information, moving frequently, and coming from neighboring countries to receive treatment and return. This population, in particular, is at high risk for loss to follow-up because of challenges to track and facilitate treatment completion. Adolescents were also considered by healthcare personnel as more challenging than adults to convince to start treatment, specifically those who had dropped out of school, were without stable employment, lacked family support, and were believed to be “unconcerned” about their health in general. Challenges due to poverty were commonly described. Precarious living conditions hindered accessing patients or attempting to track those who had abandoned treatment.

*Informal Laborers. “The problem is with the people who at times, because of challenges of work, do not want to come in to take the treatment here [healthcare center] because they say, “I need to continue to work”….They are going to choose a day job and not take the medication.” [Healthcare personnel, FG]*

*Addiction. “The people who we work with don't have means of transportation, are drug addicts, alcoholics. We have average people too but we work a lot with people that do not have means and they suffer because they are drug addicts or alcoholics that begin treatment and then abandon and therefore you have to go find them. It is a manual job, very tiring because it is hard.” [Healthcare personnel, FG]*

*“He [brother] stopped taking the treatment that the doctor from here sent him. Well, he abandoned everything and told me: “Well, I am better.” He began to drink and do drugs again.” [Patient/family member, FG]*

*Adolescents. “Family support it seems to me contributes, helps; the level of education as well. I also believe that it is a cultural question. A person who works, who has a family, who has to move ahead, this person will be cured. And the person who is uninterested or is adolescent, a kid who is 20 years old who left school and maybe some days he has a day job and other days he does not, this is the person who is not concerned with continuing treatment, nor is he consistent.” [Trained community healthcare worker, FG]*

*Living in Poverty. “All of this region (…) which is all of this zone, is a region of settlement, region of slums and areas of people who live in very poor standards of living, indigent. You go to places where you find they are almost in caves.” [Healthcare personnel, FG]*

*Staff Turnover*. Patients, healthcare personnel, and directors strongly agreed that the stability and consolidation of the healthcare team is vital to achieve treatment success. However, political changes, poor compensation, and/or intent to gain experience and move on were to blame for the high staff turnover rates and the consequent need for staff retraining. New physicians were said to gain experience in more distant healthcare centers and then “move on” when the experience was obtained or when they were offered employment with better compensation. Patients and family members recognized nurses as playing a key role in their care. Nurses were identified as the primary DOT supervisors and treatment coordinators. However, nurses described managing multiple duties (e.g., pharmacy, primary care clinic, and immunization clinic), but without recognition.

*“…regarding training, they [healthcare personnel] keep changing, the nurses change, the doctors change.” [District director, individual interview]*

*“The turnover of doctors and nurses leads to these methods [those that work] not being productive.” [Healthcare personnel, FG]*

*Political Commitment Issues. *Concerns of political commitment were described as “unofficial” designation of positions in the TB program, varied access to resources, and reassigning staff to different positions after political changes. Healthcare personnel and program directors reported willingness to contribute to TB efforts but in doing so accepted added responsibilities without additional compensation or recognition. The national TB director indicated that his position was only recently designated as an “official” post. Lack of basic resources and varying access to resources at healthcare centers (e.g., no access to computers) were described. Political changes were highlighted as important because they resulted in reassignment of healthcare personnel to different areas, causing disruption in the flow of delivery of care and requiring training of new staff members. Staffing and resource issues were perceived as compromising established community perception of quality of healthcare services at the local level and confidence, as well as requiring increased efforts and resources to retrain new staff.

#### 3.3.2. Facilitators

*Social Support*. Social support from family, friends, and healthcare personnel was considered essential to provide emotional and practical support and encouragement.

*“His friends came to my house and were there all day with him. They were with him every minute. They spoke with him, trying to get him to get out of bed and thanks to him and his friends that were with him…they lifted him up…you can say that they were friends. These are true friends. His friend was sick and it didn't matter to him….He came to see him everyday.” [Mother of patient talking about son's friend, FG]*

*Established Infrastructure. *The physical establishment of a decentralized healthcare system, although described as being underutilized, was seen as a strength to minimize the challenges of access to TB care. Each municipality managed 15 to 25 local healthcare centers located on average at every 10 to 15 blocks, with some municipalities reporting having healthcare centers that supported more rural populations with challenges of access due to limited public transportation and travel cost. Established and dispersed laboratories were reported by healthcare personnel and directors to be adequately equipped to conduct basic tuberculosis testing.

*“In our country there doesn't exist geographic inaccessibility….Here it is very rare that there is geographic inaccessibility. You have hospitals, healthcare centers, medical units, all this a part of the imagination—the person knows where to go. I don't know if they know where to go to receive better care but they know where to go…. Abandonment of treatment in Argentina is linked to places of major urban concentration.” [Local director, FG]*

### 3.4. Organizational Factors

Organizational barriers and facilitators included factors related to DOT, interventions with limited implementation, patient tracking, and perception of quality of healthcare services at the local healthcare centers.

#### 3.4.1. Barriers

*Resistance to Use/Lack of Belief in DOT. *DOT was described as both a facilitator and a barrier. Some patients felt more attended to and that their needs were more quickly addressed, but other participants considered DOT burdensome both to patients and to healthcare services. DOT was described as “intrusive” and “too demanding” and was a likely cause for some patients to return to settings where self-administration was standard. Many of the healthcare personnel and directors stated that there was a belief that those completing treatment by DOT would also complete successfully by self-administration; in contrast, there are patients who, due to challenging situations, will not complete treatment successfully no matter what intervention is used. A major challenge reported by healthcare personnel and directors was trying to convince patients who had initiated self-administration of treatment to transition to DOT once they were transferred to a healthcare center. They believed that, due to customary practices, doubts about the effectiveness of DOT, or issues of feasibility, some providers, particularly at hospitals or in the private sector, offered only self-administration monitored with periodic evaluations. Some healthcare personnel admitted that they themselves first needed to be convinced of the effectiveness of DOT through experience, rather than simply complying with standards. Once convinced, they were better prepared to recommend DOT, and their attitudes towards the strategy spilled over to other personnel and patients. Although convinced, many maintained concerns about feasibility.

*Patient Perspective of DOT. “Yes it is difficult [DOT]. It is hard to come in.” [Patient, FG]*

*“The nurse attends to us, she attends to us very well.” [Patient, FG]*

*“I don't have a problem coming to the clinic. Besides they attend to me well, the doctor and the nurse.” [Patient, FG]*

*Convincing Patients. “It is to say, practically it is not an obligation, but we try to convince them [patients] to do supervised treatment. Many ask us “why, it is hard for me to come in.” What happens is the national program indicates that it has to be supervised treatment or else they don't give us the drugs.” [Municipal program director, individual interview]*

*Need to Be Convinced. “The patient that completes DOT also completes treatment in their house.” [District director, FG]*

*“…I was here [working in the healthcare system] during the time when treatment was self-administration, period. The idea of treatment changed and I had to be convinced with numbers from where here [one healthcare center] I had patients treated by self-administration and there [another healthcare center] DOT patients and here was a percentage of abandonment very high and there a percentage of abandonment much lower; therefore I was convinced.” [Healthcare personnel, FG]*

*“…There is a resistance. It is inevitable. The primary resistance is our own until you are convinced that with the direct supervision of treatment, adherence is improved. Once you are convinced, it begins to spill over to everyone else and the patients…” [Healthcare team, FG]*

*Inefficiency in Data Collection and Management. *Data management (e.g., patient notification and tracking) was paper-based, and records were organized and stored idiosyncratically at each healthcare center. None of the healthcare centers visited had access to a computer to manage data (e.g., no electronic medical records or computerized tracking or data management systems). Moreover, the paper-based process varied by healthcare facility. For example, at the healthcare centers where DOT was implemented there was a daily monitoring sheet, and at hospital-based clinics there was a 4 × 6 card that documented start dates and dates when a patient came to retrieve the monthly supply of medication. Multiple healthcare personnel reported taking patient records home to create an organized computer-based database to more effectively manage patients.

Patient tracking, referral pathways, and outcome monitoring were reported as discontinuous or fragmented with challenges to assure arrival of transfers to other facilities or to assist in monitoring patient progress. For example, a patient may start treatment in one location, move, and then either continue or abandon treatment or fail to provide information to a new provider to get restarted on a new treatment regimen. An example highlighted was of a patient with MDR-TB who moved to another province and sought treatment. The patient was started on first-line drugs until the prior attending physician phoned later to inquire if the patient had arrived to transfer care of treatment monitoring.

Some healthcare personnel described distrust in the overall accuracy of data reported to the regional or national level. The regional level described high rates of complete treatment outcome reporting. In addition, the challenges to accurate national oversight of TB treatment outcomes were depicted as stemming from known underreporting of cases and outcomes from the private sector. Exacerbating the challenges of oversight and planning were delays of up to 2 years to produce TB reports because of the time required to report and process the paper-based data.

*Perception of Low Quality of Care. *The decentralized system's potential was recognized as not being maximized. According to the national TB director, TB cases are concentrated in centralized locations, particularly infectious disease specialty hospitals. Healthcare centers were described as varying in size, resource availability, hours of service, and staff composition. Examples provided included having one pulmonologist to attend to patients at multiple healthcare centers, resulting in continuous traveling, days in which centers were without a pulmonologist, and healthcare centers with small, crowded, general waiting rooms where TB patients were required to wait for appointments and to receive treatment. Because of the variability in clinic services and resources across the system, healthcare personnel indicated that there was a perception of a low quality of services provided at the local, smaller healthcare centers by communities and hospital staff. This perception of a low quality of services was thought to contribute to a lack of referrals of patients from a larger facility to a local healthcare center and a general lack of communication among facilities, which was considered another important barrier to patient tracking and treatment completion. Although personnel at local healthcare centers reported attempting to improve communication between centers and hospitals to in turn improve patient referrals, they indicated that these efforts often failed to produce results.

Nonetheless, the participating patients described being satisfied and “cared for” at the local healthcare centers. Healthcare personnel and directors at the healthcare centers felt they were better able to track, follow up, and return defaulters to treatment compared to larger facilities such as the hospitals.

*Variability of Services across Healthcare System. “There is one thing very important; every primary healthcare center is a different world. You [researchers] see this one. This is distinct from other centers that are 2 × 2, very small.” [Municipal director, individual interview]*

*“Not having a person, a specialist in a center is an obstacle because the person then has to be transferred to a larger center.” [Healthcare personnel, FG]*

* “There are not enough pulmonologists.” “There are not pediatricians.” [Healthcare personnel, FG]*

*“People come to be attended to at the centers with so many children in the waiting room and more during the winter when everything is closed and there is a lack of ventilation.” [Healthcare personnel, FG]*

*Second Class Care. “The healthcare team [at hospital level] do not have confidence in the ability of the health care centers. It is as if they [healthcare centers] are second class. There is this idea that the healthcare centers, because they are peripheral, they are second rate.” [Regional director, individual interview]*

*Lack of Communication among Facilities. “I am trying to incorporate programs at the provincial hospitals but it is difficult because they [hospital personnel] do not want to. We were able to convince one hospital (name removed) to start this year to pass the hospitals statistics because it has a 35% rate of treatment abandonment, which is why we have currently high rates of tuberculosis and multiresistance. We have lots who [patients] abandon. They [hospital staff] do not notify us; therefore, we cannot go to look for them.” [Municipality director, FG]*

*“Within this district we have three hospitals (names removed). The hospitals (…) do not refer patients to us. They stay there, they manage them, and it is where we have the highest rates of abandonment because they [hospital staff] do not go out to look for patients [who abandon treatment].” [Healthcare personnel, FG]*

*Reliance on Personal Commitment. *Daily work in the TB program was described as largely based on personal commitment, often “beyond duty,” rather than on program structure. Healthcare personnel emphasized that, without their personal commitment, stemming from both personal and external expectations, the program would not accomplish the current results. Examples of personal commitment included use of personal funds to cover expenses for which no resources were assigned and no official budget existed, such as providing breakfast to encourage medication adherence for those who did not have money for proper nutrition, and using personal cars and covering gas expenses to attend community events, make home visits, or travel to healthcare centers for supervision (directors).

*“We are the firefighters of medicine risking our life voluntarily.” [Municipal director, individual interview]*

#### 3.4.2. Facilitators

*Individualized Flexible Treatment. *As previously described under Modes of Treatment Delivery, flexibility and negotiation with patients to keep them in treatment was seen as a supportive method, an incentive for compliance, and a means to lessen challenges to coming in daily to receive treatment. Although the availability of antituberculosis medication was reported as a problem in the past, healthcare personnel and directors indicated that it was not an issue during the time the interviews were conducted. Additionally, maintaining continuity and stability of healthcare personnel was recognized as an important factor to promote patient and community perception of quality of healthcare services at the local level.

*Interventions with Limited Implementation. *Interventions limited to one or a few locations were implemented to address some of the identified barriers perceived within their municipality. For example, one of the healthcare centers provided DOT on a walk-in basis (without appointment) and through a separate door around the side of the building. The TB patients were rerouted away from the full waiting rooms. Other program directors reported having a number of healthcare centers open 24 hours a day to facilitate access to treatment for patients. Some municipalities reported utilizing local politically appointed community advocates to locate patients who had abandoned treatment—to go to the patient's home and encourage the patient to return to treatment if other attempts to return the patient to treatment had failed. They also described training community healthcare promoters to provide information and support and facilitate referrals within their communities. One healthcare center reported training DOT observers (e.g., a night guardsman) to provide DOT to patients outside of hours of attention to facilitate treatment delivery.

*“The problem is when they have to go to work. How do they do it? People go far to work and therefore come early. The healthcare center is not open, except those that are open all night which are few…therefore the people leave before and return after [clinic closes]. This is a problem.” [Municipal director, individual interview]*

*Financial Subsidy. *Some patients with TB qualify for a government subsidy to offset the financial burden of the disease. The government subsidy was seen by interviewees as an incentive to continue treatment, but healthcare personnel noted that the incentive was undermined by confusing and not well-understood subsidy regulations, leading to low rates of application, and by administrative delays upwards of 6 months following treatment initiation. In addition, it was noted that the subsidy must be initiated by the attending physician, who may not have been the individual who started the paperwork. If the application is not initiated within the first 2 months of treatment, patients lose their opportunity to apply. Regional reports indicated that about 10% of TB patients in Region V were receiving the subsidy. Even though the delay is substantial, it is a marked improvement from the reported historic wait of up to 3 years.

*“…people live day by day here. It's not like work will wait six months or nine months when they are better. They are waiting for work….They have to go out and look for money otherwise they do not eat….There is subsidy for tuberculosis that they are paying now, but it arrives at best six months after completing treatment.” [Healthcare personnel, FG]*

## 4. Discussion

To our knowledge, this was a first of its kind qualitative study assessing modes of treatment delivery and barriers and facilitators to TB treatment success from multiple perspectives in Argentina. This research served as a foundational evaluation for a study currently in progress to assess patient and system factors associated with successful treatment of tuberculosis. However, evidence from this study may also identify—for both policy makers and healthcare personnel dedicated to TB management—weaknesses within the system and interventions to strengthen the system. Findings highlight that many of the barriers to treatment success were at the system/organizational level, but an interplay of personal and structural/social factors also influenced treatment outcome. Interventions were in place in some districts to counter some of the perceived barriers. The facilitators primarily focused on support (individual and community), flexibility, commitment, and continuity of care.

### 4.1. Modes of Treatment Delivery

Our findings highlight a potential discrepancy between the reported mode of treatment delivery as DOT and the mode of delivery in practice. We found that DOT was often considered a component of a larger support package and more “flexible” variations were in place to meet patients' needs and situation. Weekly or bimonthly treatment monitoring is, in effect, self-administration. Recent studies have demonstrated that the degree to which treatment is directly supervised can vary between and within countries [28–31]. Reports issued by the government of Argentina also highlight that DOT is not widely implemented in some provinces, especially those with the highest case loads and largest TB burden [5]. The low rates of DOT application could also be related to not all healthcare personnel being convinced that strict DOT was necessary and challenges to convincing patients to start daily DOT at a healthcare center when they had previously received treatment by self-administration from an outpatient hospital-based clinic.

### 4.2. Individual Factors

Our study highlighted the importance of establishing strong patient-provider relationships to facilitate treatment success, which has been previously described in the literature [14, 31, 32]. However, presumably the development of a strong patient-provider relationship cannot be readily established during self-administration of treatment alone. We found that communication and established relationships with healthcare personnel helped patients combat fear of TB and increased their knowledge of the disease. Stopping treatment due to feeling better has been identified as the cause of nearly 30% of patients abandoning treatment in a study conducted in Zambia [33]. Patients and family members of patients in this study described how challenging it was to convince individuals with TB to continue treatment when they felt better. In this study, most patients were positive about their experiences at their local healthcare centers and were willing to comply with treatment because of an established trust or positive perception of quality of healthcare services at the local level. However, maintaining established community positive perception of quality of healthcare services was described as being undermined by frequent staff turnover or political changes leading to position transfers, both of which affected the continuity of local healthcare teams.

### 4.3. Structural/Social Factors

Adequate individual and community awareness of TB was considered by patients and healthcare personnel to promote adherence, decrease stigmatization, and improve TB outcomes. Delays in disease detection can result in more advanced and complicated cases. Our findings highlight the need to increase TB knowledge in communities, particularly in schools and among healthcare providers, to address the misconceptions about the disease and lessen the stigma, as well as decrease diagnostic and treatment delays. What were termed “health tours” by some participants in this study exemplified the challenges to treating and tracking mobile populations. In this study, participants understood that TB was curable. Patients acknowledged that fears were diminished when healthcare personnel informed them about the disease, and healthcare personnel recognized the importance of explicitly informing patients that TB is curable only by completing a full course of treatment. Other researchers have reported similar factors impacting TB treatment success: stigma and fear [34–36], nonsalaried employment, fear of losing employment or the opportunity to work [36, 37], challenges of mobile populations [38], and misconceptions and lack of knowledge about TB and its treatment [14, 39–41].

### 4.4. Organizational Factors

Adherence to treatment is the responsibility of both the patient and the system; however, the system should facilitate compliance. We identified patient motivation to adhere to treatment and healthcare personnel commitment to the patient as important factors contributing to TB treatment success. Because motivation is difficult to operationalize, and other important influences may be overlooked, Munro et al. [14] warn about attributing personal motivation to treatment adherence. Our findings highlight an organizational reliance on personal commitment of healthcare personnel who: provided food to patients to promote treatment adherence, used personal funds to cover TB treatment-related expenses, and were creating individual databases to better manage and track patient treatment.

The support package, not described as such by participants, included strong patient-healthcare personnel relationships, assistance with applying for financial subsidy, provision of food, and other provider-patient interactions. Patient-centered approaches, individualized support and monitoring of treatment adherence, use of incentives to continue treatment, and interventions to return patients who abandon treatment have been reported in the literature to improve TB outcomes [4, 42–44]. We found that a governmental financial subsidy for patients meeting requirements had been established, but administrative delays in distribution lessened the impact of the subsidy. Providing a subsidy early would help patients who are the most likely to abandon treatment.

Findings from this study suggest strengthening and better utilizing the established decentralized system. Ideally, hospitals in a decentralized system would be utilized for complicated and difficult cases, and healthcare centers would primarily focus on dispersed TB cases. Decentralization of treatment and care has been reported to improve treatment outcomes by minimizing travel cost and distance to access healthcare [45, 46]. In some countries access to health services has been reported to be a major barrier [14, 31, 47, 48]. In this study, in contrast, the healthcare centers were reported to be widely dispersed (estimated at one about every 15–20 blocks) and access to care was not considered the major barrier, although some participants cited having districts with more rural populations. Of more concern was a perceived low quality of healthcare services provided at the smaller community healthcare centers. Many healthcare workers believed the perceived low quality of care led patients to travel further to larger facilities and prevented healthcare personnel from referring patients to local healthcare centers where treatment monitoring could be conducted closer to where the patients lived and where there were fewer cases, making tracking and returning patients who default back to treatment easier. The resulting TB concentration at the larger facilities, where self-administration of treatment was the usual care, was considered a major contributor to patients becoming lost in the system due the difficulties managing, tracking, and returning patients who abandoned treatment. The concentration of cases at larger facilities was, in part, the result of major disparities in the quality of the facilities and services provided at local healthcare centers, fueling the perception of a low quality of healthcare services at the local level and ultimately the underutilization of the established decentralized system. The best methods to strengthen the smaller healthcare centers would need to be identified prior to inundating these centers with TB patient referrals.

### 4.5. DOTS/DOT

Reflecting on the five DOTS components (securing political commitment, strengthening detection and diagnosis, ensuring drug availability, monitoring outcomes, and providing directly observed therapy (DOT)), a number of our findings highlight persistent operational problems in its implementation. Political commitment to the TB program was questioned with findings, such as lead positions (e.g., the national TB director, regional directors) not officially recognized, standard shifting of personnel with political changes, and varying quality of services offered at the local healthcare centers. With regard to detection and diagnosis, it was recognized that a major barrier was a lack of consideration of potential TB diagnosis (low index of suspicion of TB) by individuals, community, and healthcare professionals, which impacted treatment seeking and delayed diagnosis. Although a regular drug supply was reported at the time of the study, Argentina experienced a medication shortage at the time of the paper's preparation. In Region V, some of the first and second line antituberculosis medications were unavailable for an extended period of time.

Data management was paper-based and was idiosyncratically organized by the healthcare centers. No healthcare center visited had access to a computer to help manage caseloads. This lack of an integrated computerized system to manage patient data may contribute to the high numbers of identified TB cases not having final treatment results (27% according to the latest national TB treatment results from 2009) [49] and monitoring and evaluation delays, which can lead to programmatic failure to respond to poor outcomes. During the duration of the study, an online reporting system, used by regional directors to input paper-based patient data, was being implemented in some provinces, but it was not being used for patient tracking and follow-up at the local level. The impact of the online reporting system has yet to be evaluated. Expanding the current web-based national system to include individual patient tracking and treatment monitoring could decrease the number of cases missing final treatment outcomes and aid healthcare personnel in managing their caseloads.

Lastly, DOT has been alternatively viewed as a supportive model of care and a control model of care that likely decreases responsibility for patient self-care [43]. Our findings suggest that patients who were receiving treatment by DOT believed they were cared for, but many healthcare personnel indicated, in their opinion, that those who completed with DOT would also complete by self-administration. Overall, the DOT strategy was perceived as an effective tool for treatment success, but not sufficient in and of itself. Other factors, such as education, TB knowledge, and socioeconomic situation, were considered more influential. More recently, the effectiveness of DOT has been questioned [37, 42, 43, 50–52]. Instead of a dogmatic approach that insists DOT is the* only* technique to assure effective treatment, DOT is now listed as an example of a* possible* measure to assure and aid in treatment adherence [4]. Congruent with this shift to meet patient-centered needs and offer individualized support, we identified multiple examples of flexible patient-centered approaches such as negotiated number of DOT days, no-wait treatment, training a night guardsman, and 24-hour healthcare centers. Despite the potential benefits, we did not find evidence of patients selecting their own DOT treatment supervisor. Interventions for TB care should be standardized but also allow some flexibility based on the needs of the individual and local healthcare center.

### 4.6. Limitations

We believe the barriers and facilitators identified in this study provide valuable insights from multiple perspectives into factors impacting TB treatment success in high TB burden regions of Argentina. However, there are some important limitations to mention. Although results may not be generalizable to the entire country, the public healthcare system in other regions of Argentina is governed by similar political and organizational structures. The inclusion of healthcare personnel was through purposive sampling of healthcare municipalities with higher and lower rates of treatment success based on historic records and recommendations of the regional TB director. We interviewed those involved in TB efforts at the selected sites. However, for patient/family participants, we relied on healthcare staff to approach and invite patients to participate. Therefore, we do not know the number of patient/family participants who were invited, declined to participate, or did not show up for the group interviews. Those who agreed to participate in the study may have been those most adherent to their TB treatment. Although we requested that healthcare staff attempt to invite patients who had abandoned treatment, this did not occur. We were, however, able to include testimonies of the family members of such patients and the healthcare professionals who had been responsible for the care of these patients. Unfortunately, some of the audio recordings of the patient/family interviews were difficult for transcriptionists to decipher and sections of recordings were not transcribed, which resulted in fewer direct patient quotes. Lastly, the interviews were focused on healthcare teams at healthcare centers and at the municipal level; therefore, healthcare personnel in hospitals were not interviewed. However, regional directors were able to speak to the process of how patients were managed at the regional hospitals.

## 5. Conclusions

To make substantive changes in countries where TB treatment success is consistently low and rates of drug resistance are increasing, the investigation and identification of root causes is paramount. Achieving treatment success is inherently multifaceted and cannot be attributed solely to patient characteristics—responsibility lies with the individual and the system. Overall, the healthcare system appeared to rely heavily on personal commitment of both patients and healthcare personnel. Adherence, from patient and healthcare personnel perspectives, was often not a free choice but rather a reflection of behaviors conditioned by the sociocultural and economic context. Identifying the majority of barriers at the organizational level highlighted the importance of strengthening system-level initiatives. Interventions such as quick access to treatment through separate doors, having healthcare facilities open for extended times, and providing incentives or utilizing politically appointed community advocates had limited implementation. Increasing dissemination of TB information to the public and healthcare personnel could help reduce the stigma of TB and thereby decrease delays in diagnosis and treatment. A strengthened political commitment is needed to motivate, distribute, and support competent healthcare personnel throughout the decentralized system, minimize healthcare personnel shifting/turnover during political changes, more quickly allocate treatment subsidies to patients, and improve the accuracy and efficiency of patient monitoring/tracking (e.g., centralized patient tracking system). More uniform staffing and resources across the healthcare services could promote a positive perception of the quality of healthcare services provided at local healthcare centers and improve the utilization of the established decentralized system. Flexible patient-centered care is needed to promote strong patient-healthcare personnel relationships and provide support to patients, especially those concentrated at the larger healthcare facilities receiving treatment by self-administration. Ultimately, recognizing and responding to weaknesses in the healthcare system and tailoring delivery of healthcare to patient needs rather than having patients adapt to the models in existence could impact TB treatment outcomes. Findings can be used to tailor programs to improve TB treatment outcomes in similar settings.

## Figures and Tables

**Table 1:** Barriers to and facilitators of successful completion of treatment by category.

	Barriers	Facilitators
Individual	-Drug side effects (e.g., GI upset, bitter taste) -Lack of TB knowledge about the disease and treatment -Fear related to TB (e.g., incurable, loss of work, or discrimination) -Interpretation of feeling better means cured -Comorbidities (e.g., alcoholism, drug addictions) -Personal/family challenges	-Desire to be cured, personal motivation -Personal experience with other TB patients -Strong patient-provider relationship -Personal characteristics of healthcare personnel: committed, compassionate, supportive, able to establish trust (builds rapport with patients and longevity in the community and center), having personal calling to serve others, and able to humanize disease and situation -Interpreting feeling better as cured

Structural/Social	-Access to healthcare centers (e.g., distance, transportation issues, and cost) -Poverty, precarious living conditions -Low wages for healthcare workers -Long treatment course -Vulnerable patient populations -Informal employment (e.g., day labor), women with childcare challenges, “health tours,” with comorbidities (e.g., addictions, HIV/AIDS), living in poverty, adolescence	-Dispersed healthcare centers throughout communities/region -Free-of-charge medication and services for TB treatment -Social support of family and friends, healthcare personnel, volunteer community health promoters
	-Discrimination and/or stigmatization -Lack of education in communities and schools leading to poor TB awareness and understanding of treatment -Perception of low quality of care offered at healthcare center -Instability of political commitment/support -Financial subsidy delays and low rates of application due to inadequate dissemination and clarity of policy/regulations -Reassigning positions/frequent staff turnover -Lack of official recognition and monetary compensation of TB positions	

Organization/System/Health Service	-Resistance to use directly observed therapy (DOT) by some healthcare personnel -DOT -Self-administration standard at hospitals (conflicting messages to patients) -Self-administration offered first -Low index of suspicion of TB resulting in diagnostic delays -Underutilization of decentralized healthcare system -Lack of collaboration/referrals between hospital and healthcare centers -Cases concentrated for treatment at hospital level -Lower treatment success and high rates of abandonment at hospital level -Disparity in size, resources, hours of service, and staff composition at healthcare centers (e.g., short on TB supplies, no computers, and lack of specialists or physicians) -Overburdened staff -Inefficiency in collection of data (outcome monitoring) -Patients lost to follow-up, poor tracking -Paper-based healthcare records (no computers at centers) -Lack of centralized surveillance system -Delayed and underreported case outcomes→delayed/incomplete program evaluations (up to 2 years) -Mistrust in accuracy of reported data	-Subsidy for those who continue/complete treatment -Being convinced of DOT effectiveness -DOT -Decentralized healthcare system -Healthcare centers situated at about every 10–15 blocks -Facilitating healthcare center characteristics (limited implementation) -Open 24 hours -Provision of DOT without appointment and through separate door (not having to wait in waiting room) -Use of politically appointed community advocates to find and return patients to treatment -Medication availability (not always the case) -Established laboratory/diagnostic network considered reliable and available -Continuity of healthcare personnel -Capacity of TB healthcare team members
